# Torque Ripple Minimization of the Permanent Magnet Synchronous Machine by Modulation of the Phase Currents

**DOI:** 10.3390/s20082406

**Published:** 2020-04-23

**Authors:** Cezary Jędryczka, Dawid Danielczyk, Wojciech Szeląg

**Affiliations:** Institute of Electrical Engineering and Electronics, Poznan University of Technology, 60-965 Poznan, Poland; dawid.p.danielczyk@doctorate.put.poznan.pl (D.D.); wojciech.szelag@put.poznan.pl (W.S.)

**Keywords:** permanent-magnet synchronous motor, torque ripple minimization, finite element analysis

## Abstract

This paper deals with the torque ripple minimization method based on the modulation of the phase currents of the permanent-magnet synchronous motor (PMSM) drive. The shape of the supply current waveforms reducing the torque ripple of the machine considered was determined on the basis of finite element analysis (FEA). In the proposed approach, the machine is supplied by a six-leg inverter in order to allow for the injection of zero sequence current harmonics. Two test PMSMs with fractional-slot concentrated windings (FSCW) and surface-mounted permanent magnets (SPMs) have been examined as a case study problem. Wide-range fractional analyses were performed using developed numerical models of the electromagnetic field distribution in the considered machines. The results obtained show that the level of torque ripple in FSCW PMSMs can be effectively reduced by the modulation of the phase currents under the six-leg inverter supply.

## 1. Introduction

It can be stated that the progress in the development of electrical machines (EMs) in recent decades has mostly been related to the improvements and the progress made in the development of the materials used in their magnetic circuits—like permanent magnet materials with high magnetic energy density and low-loss electrical steel sheets, or soft magnetic composites—allowing for the operation of electrical machines at higher frequencies. On the other hand, the dynamic progress in computational methods and the power of computers has allowed for precise analysis of electrical machines’ performance. In particular, the Finite Element Method (FEM) is one of the widely used methods employed for determining electromagnetic field distribution, which has led to progress in the design methods of EMs. The availability of the professional tools for the combined analysis of coupled electromechanical and thermal phenomena allows for the “optimal” design of electrical machines. It can be stated that “state of art” design methods and tools for the analysis of electrical machines’ performance allow us to synthesize construction characterized by high efficiency and/or power density and/or low cost of production, properties which should be treated as goals or constraints during the design process. Currently, besides the above discussed figures of merit, the quality of energy conversion in electrical machines is becoming a point of interest for many research and design teams. The reduction in unwonted phenomena related to energy conversion processes—like the influence on the grid (higher distortion of currents or voltages in the case of generators), noise and vibration, as well as a reduction in torque ripples—is within the scope of research activities and efforts taking place in the design stage of modern EMs. This paper is focused on one of the discussed aspects of energy conversion quality, i.e., torque ripple minimization in permanent-magnet synchronous motors (PMSMs). The need to provide high quality energy conversion in electrical drive systems is obvious. Lowering the value of the electromagnetic torque ripple decreases the levels of vibration and noise, as well as extending the life expectancy of the drive system. In many direct drive applications, such as elevator propulsion units, providing high quality torque (without the torque ripples) is essential. Therefore, reducing the torque ripples in electrical drive systems has been a point of interest for many research and design teams in recent decades. In this type of research, two major trends can be observed: (1) the reduction in torque ripples by the control system side [1,2,3,4,5] and (2) the improvement of the torque quality at the design stage by the optimization of the electromagnetic circuit of the electrical machine. In the first trend, the current harmonic injection is one of the commonly applied techniques [1,2,3]. Due to the technical difficultly of directly measuring the torque ripple in such approaches, torque observers are usually employed [2]. To reduce the torque ripples, harmonics are injected into the control system at the level of the rotary reference currents; this is mainly done by modifying the torque producing current *i_q_*. To find the *i_q_* harmonics that will reduce the torque ripples, many different optimization techniques and algorithms are used. For example, the Cuckoo behavior-based algorithm has been successfully employed using the method described in [1], while the neural network (NN)-based parameter estimators have been applied to reduce the torque ripple in motor drives using the approach described in [2]. Nevertheless, the model-based predictive current control methods suffer from a need for either complex algorithms for model parameter identification or a high amount of data for training the NN [6]. Consequently, problems of the reliability of the measured input data and the desired high control loop frequency arise.

On the other hand, the origins of the fluctuations in electromagnetic torque values lie inside the machine. In general, the torque level, as well as it ripples, are effects of the Maxwell stress tensor distribution inside the air gap of the machine, which depend on the interaction between the magnetic field’s temporal and spatial distributions excited by rotor magnets and the magnetomotive force (*mmf*) of the temporal and spatial distributions, which is generated by the winding. Therefore, the second group of studies tend to minimize the torque ripple and focus on the design of the machine’s magnetic circuit. Among others, these methods were based on the stator and rotor skewing, the profiling the magnetization of the permanent magnets, as well as introducing non-uniform air gap lengths or increasing the number of phases employed in order to suppress the cogging torque, as well as to reduce the torque ripple level of the machine [7,8].

**Figure 1:**
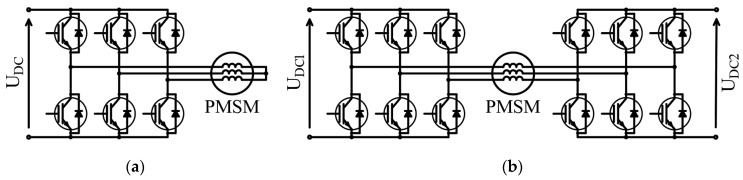
Conventional 3-leg inverter driving a permanent-magnet synchronous motor (PMSM) (**a**) and dual-fed open-ended winding PMSM (**b**).

The study presented in this paper is in some ways rooted in both of the above discussed trends in the research. As discussed in [9], the reduction in torque ripples by supply current shaping has already been investigated by, among others, Razek in 1990 [10]. In terms of the torque ripple minimization methods given in [9], the difficulties in practical realization have been pointed out as being the main disadvantages of such methods. It should be noted that the injection of 3rd integer multiplier harmonics (triplen harmonics) is not possible in PMSMs driven by conventional three-leg (see Figure 1a) inverters when the phase windings of the machine are arranged in a star connection. On the other hand, due to the decreasing cost of electronic power switches, the six-leg inverters are now an interesting alternative to three-leg systems in terms of increased reliability [11,12,13]. The application of knowledge from the literature regarding the dual-fed open-ended winding PMSM (see Figure 1b) enables the development of new, more effective methods for improving the energy conversion quality in electrical machines (by modulating the phase currents by higher harmonics and, in particular, by injecting zero-sequence harmonics).

## 2. Torque Ripple Minimization of PMSM by Current Modulation Method

In contrast to other approaches exploiting phase current modulation, in the proposed method, to find the amplitudes and phases of the current harmonics that will allow us to improve the energy conversion quality in the PMSM, the precise assessment of the torque ripple is carried out by an analysis of the magnetic field distribution, determined using FEM. Numerical models based on FEM, due to the high reliability resulted from solving partial difference equations describing the magnetic field in space and time domains, are now commonly used at the design stage of EMs [14,15,16,17]. In general, exploiting FEM-based models allows researchers to avoid building a high number of expensive prototypes, which would otherwise be needed to verify the design process. In the studied problem of torque ripple minimization, the “optimal” shape of the phase current waveforms could also have been determined by using simpler and less computationally expensive analytical methods, allowing the torque ripple level assessment to take place. Nevertheless, to superimpose the rotor and stator fields (formed by each phase current) as proposed in the field reconstruction method [18], for example, the magnetic saturation effect must be neglected. Consequently, the resulting current waveforms may not minimize the torque ripple level significantly [19]. To mitigate the above discussed problem in the proposed method, the calculation of the torque ripple factor (treated as a goal function for the current waveform shape optimization process) of the two components of the instantaneous electromagnetic torque of a PMSM are determined [20] on the basis of the finite element analysis (FEA) of the machine:

(1) $$T\left(t\right)=T_{0}+T_{r}\left(t\right)$$

where *T*_0_ is the constant (average) component and *T_r_* is a periodic component dependent on time or the electrical angle. 

There are several, known from the literature, definitions of the torque ripple factor (*T_RF_*) which are used [20] in the analysis of the torque quality of electrical machines. In the most commonly applied approach, the torque ripple factor is calculated as the difference between the maximum and the minimum value in torque *T*, when related to its average value. However, in practice, when the torque is measured or calculated in a discrete manner (e.g., in the proposed method exploiting FEM) the proper determination of the maximum and minimum values of the torque is difficult. To mitigate the above discussed problem, in the presented work, the torque ripple level is expressed by the root mean square (RMS) value of *T_r_*, related to its average value, *T*_0_:

(2) $$T_{RF}=\frac{T_{r}{}_{RMS}}{T_{0}}\cdot 100\%$$

where *T*_0_ and the RMS value of *T_r_* are calculated for an electrical period of supply voltage at the steady-state operation of the studied machine.

To determine the value of the instantaneous electromagnetic torque *T*(*t*) of a PMSM, the integral of the tangential *f_t_* component of the Maxwell stress tensor, over the cylindrical surface of the radius *r* and length *L_i_*, placed in the air gap of the machine is calculated:

(3) $$T\left(t\right)=rL_{i}\int_{0}^{2\pi }f_{t}\left(t,\vartheta \right)d\vartheta$$

where the angle ϑ describes the location of the *d*ϑ on the circumference of the surface.

The tangential component of the Maxwell stress tensor *f_t_* is expressed by the normal (*B_n_*) and tangential (*B_t_*) components of the magnetic flux density vector **B** in the air gap of the machine, calculated by the FEM:

(4) $$f_{t}\left(t,\vartheta \right)=\frac{1}{\mu_{0}}B_{n}\left(t,\vartheta \right)B_{t}\left(t,\vartheta \right)$$

In general, the distributions *B_t_*(ϑ) and *B_n_*(ϑ) can be represented by complex periodic functions that depend upon the magnetic circuit structure of the machine, i.e., the number of poles, the number of stator slots, the magnetization pattern and geometry of the magnets, and the geometric and magnetic properties of the stator. Additionally, they are also time-dependent functions defined by winding structures and their phase currents. The interaction between the temporal and spatial distributions of these functions contributes to the generation of the average *T*_0_ and pulsating *T_r_* electromagnetic torque components. Despite the high computational complexity, only the application of the numerical FEM model on the machine allows all of the abovementioned dependencies for torque ripple level assessment to be taken into account. 

In the proposed approach, it is assumed that the machine is supplied by the phase current waveforms, represented by this sum of the harmonics:

(5) $$i_{k}(t)=I_{m1}\sin\left(\omega t+\left(k-1\right)\frac{2\pi }{3}\right)+\sum_{n=3}^{l}I_{mn}\sin\left(n\left(\left(\omega t\right)+\left(k-1\right)\frac{2\pi }{3}\right)+\varphi_{n}\right)$$

where *k =* 1, 2, 3 for the phase a, b, c, respectively, *n* is an odd integer greater than 1, *l* is the maximum harmonic order taken into consideration, φ*_n_* is the phase of the *n* harmonic and *I_m_*_1_ is the amplitude of the fundamental harmonic of the phase current. 

The amplitudes *I_mn_* are defined by their percentage *I_Pn_* content in relation to the *I_m_*_1_:

(6) $$I_{mn}=\frac{I_{Pn}I_{m1}}{100\%}$$

In order not to introduce any asymmetry into the magnetic circuit, the even harmonics of the current waveform were assumed to be equal to zero in the presented studies. 

The values *I_Pn_* and φ*_n_* for considered harmonics can be represented as a components of vectors **I_P_** and **φ**, which sought next to minimize the *T_r_* component of (1). The goal function used in the process of finding the optimal “shape” of the current waveform has the following form:

(7) $$F_{g}\left(Ip,\varphi \right)=\frac{T_{RFh}\left(Ip,\varphi \right)}{T_{RFr}}$$

where *T_RFh_*(**I_P_**,**φ**) is the value of the torque ripple factor calculated for injected harmonics **I_P_**, **φ**, and *T_RFr_* is the torque ripple factor calculated for the reference case, i.e., a machine supplied by the fundamental harmonics of the phase currents.

The components of vectors **I_P_**, **φ** can be treated as the design variables for the optimization process. Since the number of design variables is high (for example, equal to 18 considering *l* = 19) and the goal function call is quite expensive in terms of computation time (about 20 min for each goal function evaluation), in the proposed method, the process of finding the optimal current shape is performed in two main stages. Firstly, the values of particular harmonic phases are each determined for the assumed values of their amplitudes. Next, the values of the amplitudes are searched for using sequential parametrical calculations to narrow the design space. 

The proposed method of torque ripple minimization using current modulation has been explained in more detail and demonstrated in the case study problem described in Section 3. The PMSM of fractional-slot concentrated windings (FSCW) was intentionally selected for the case study. Despite the many advantages compared to machines with distributed windings (including lower winding cost and simpler manufacturing technology), the FSCW PMSMs suffer from parasitic effects caused by the high distortion of the magnetomotive force distribution [21]—in particular, increased eddy current losses in the rotor conductive elements, as well as an increased level of torque ripples.

## 3. Case Study Problem

**Figure 2:**
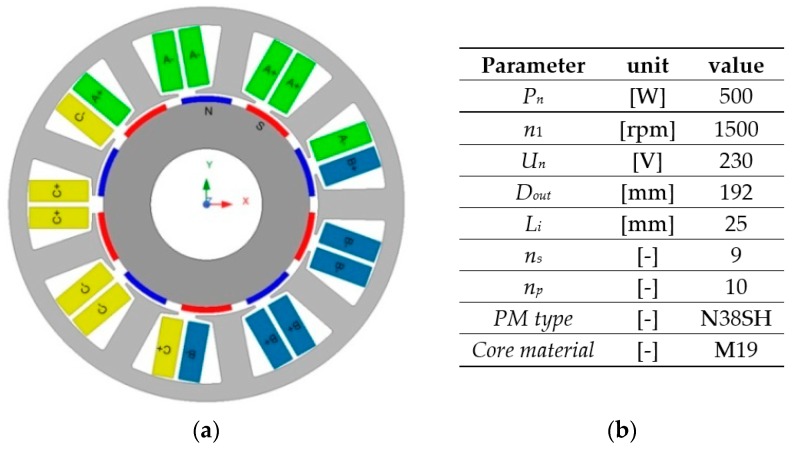
Structure of the magnetic circuit (**a**) and major parameters (**b**) of studied PMSM.

Next, the proposed method has been applied to demonstrate the effect of harmonic injection on an FSCW PMSM with a surface-mounted permanent magnet (SPM) rotor. The structure of the magnetic circuit and the main parameters of consideration in the case study problem machine are shown in Figure 2. A tested PMSM consists of nine stator slots (*n_s_*) and 10 magnetic poles (*n_p_*). Due to the high value of the least common multiple of *n_p_* and *n_s_*, the value of the cogging torque is low. Moreover, the shape and dimensions of the magnetic circuit in the machine are initially optimized by FEA in order to reduce the torque ripple level.

## 4. Numerical FEM Model of PMSM and Results of Simulations

**Figure 3:**
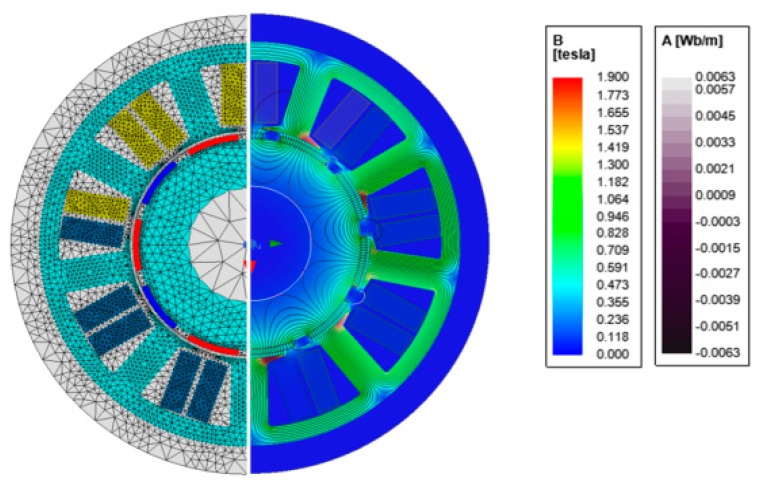
Applied FE mesh and exemplary plot of magnetic flux density.

To assess the impact of current harmonics on the level of electromagnetic torque ripples, the numerical model of studied PMSM was developed in the ANSYS Maxwell environment. The considered domain was subdivided into about 25,000 triangular elements. The applied finite element (FE) mesh and exemplary magnetic flux density distribution are shown in Figure 3. A time-stepping algorithm was employed and the period *T_f_* of the fundamental harmonics was divided into 1500 steps. Equations (2) and (5) were implemented in the developed model in order to perform a current waveform shape optimization.

### 4.1. Optimization of the Current Waveform Shape

**Figure 4:**
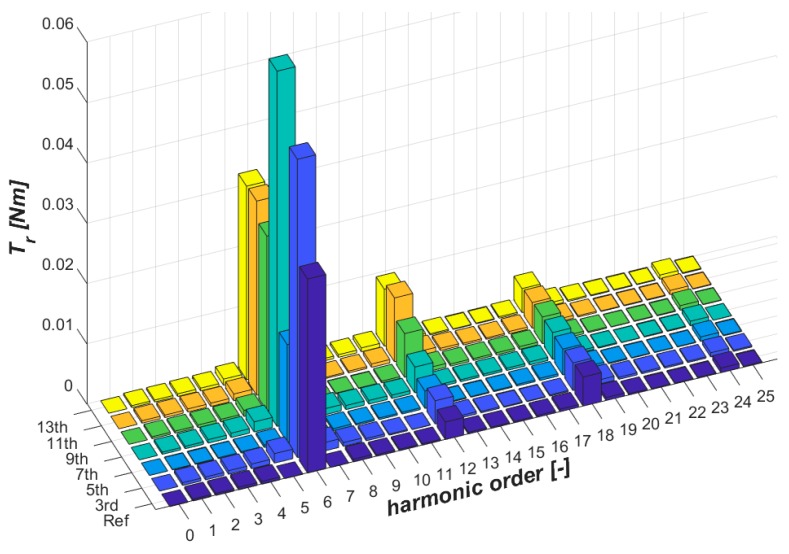
Harmonic content of torque ripple for odd harmonic injection.

The influence of particular phase current harmonics was examined first; the harmonics of 3rd, 5th, 7th, 9th, 11th and 13th orders were then injected into the phase current waveform. The calculations were performed for the rated value of fundamental current harmonic, arbitrary set values of amplitudes and different phases of particular current harmonics. The maximum torque per ampere control scheme was assumed. To study the impact of the injection of the harmonics, the Fast Fourier Transform (FFT) method was performed on the calculated *T_r_* waveforms. The results of the performed FFT of the torque ripple waveforms are illustrated in Figure 4.

When comparing the obtained results to the referenced case (the machine was supplied by the fundamental harmonic denoted as *Ref* in Figure 4), it can be seen that all the studied harmonics have an impact on the torque ripple level. Nevertheless, only in the cases of the 5th, 9th and 13th harmonics was a reduction in the amplitudes of particular torque ripple harmonics observable. At this first stage, the proper phases of these particular harmonics were searched for.

**Figure 5:**
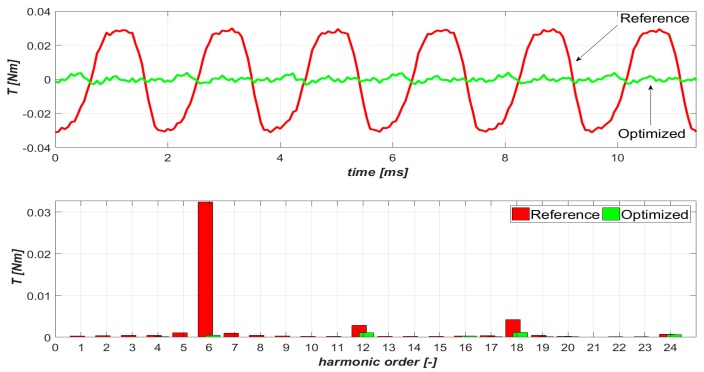
Torque ripple waveforms and their harmonic content (base structure of *n_s_*/*n_p_* equal to 9/10).

Next, parametrical analyses were conducted to find the optimal (in terms of torque ripple level) amplitudes of the particular harmonics. The odd harmonics up to the 19th order were all considered. The torque waveforms were determined by FEA for over four thousand different combinations of values of odd harmonic amplitudes. Finally, after narrowing the search space, the following values of harmonic amplitudes were determined: *I_P_*_3_ = 16.3, *I_P_*_5_ = 1.125, *I_P_*_7_ = 1.125, *I_P_*_9_ = 3.31, *I_P_*_11_ = 0.075, *I_P_*_13_ = 0.15, *I_P_*_15_ = 0.24, *I_P_*_17_ = 0.08, *I_P_*_19_ = 0.24 in % of *I_m_*_1_. The determined torque ripple waveforms *T_r_* and their harmonic content for fundamental (reference) and optimized current harmonics (optimized) are shown in Figure 5.

When comparing the torque values of the ripple factor *T_RF_*, calculated for the machine supplied by reference sinusoidal current (0.65%) to the value of *T_RF_* (which was obtained for the optimized current shape (0.04%)), it can be concluded that the proposed current harmonic injection method will allow the torque ripple level to be reduced by more than 16 times.

To verify the proposed approach for reducing the torque ripples by using the current harmonic injection, the above optimization calculations were repeated for the second structure of PMSM. The machine with the same stator geometry and an *n_p_* value equal to eight was examined. The winding layout was also changed. The determined values of the harmonic amplitudes for the second structure are as follows: *I_P_*_3_ = 10, *I_P_*_5_ = 0.75, *I_P_*_7_ = 1.05, *I_P_*_9_ = 2.4, *I_P_*_11_ = 0.05, *I_P_*_13_ = 0.05, *I_P_*_15_ = 0.15, *I_P_*_17_ = 0.05, *I_P_*_19_ = 0.15.

**Figure 6:**
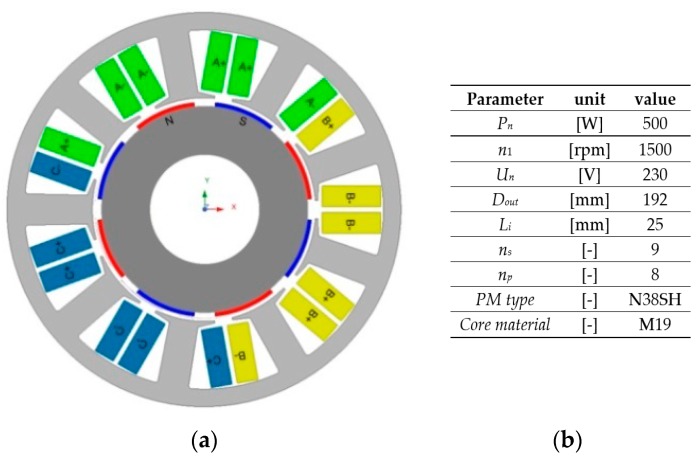
Structure (**a**) and major parameters (**b**) of studied PMSM (second structure of *n_s_*/*n_p_* equal to 9/8).

**Figure 7:**
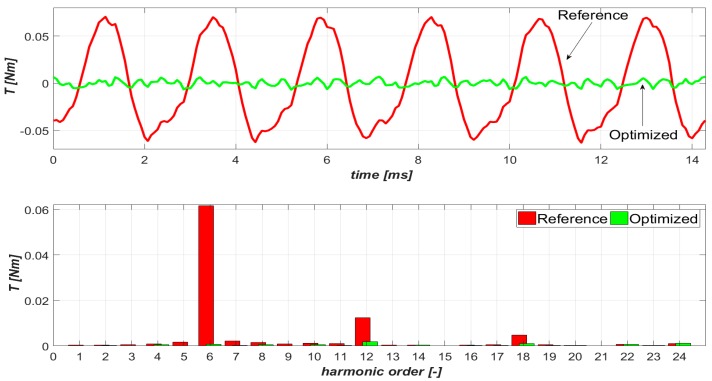
Torque ripple waveforms and their harmonic content (second structure of *n_s_*/*n_p_* equal to 9/8).

The structure of the PMSM that was examined second is shown in Figure 6, while the determined torque ripple waveforms *T_r_* and their harmonic contents for fundamental (reference) and optimized current harmonics are shown in Figure 7.

### 4.2. Influence of the Current and the Torque Angle Values

**Figure 8:**
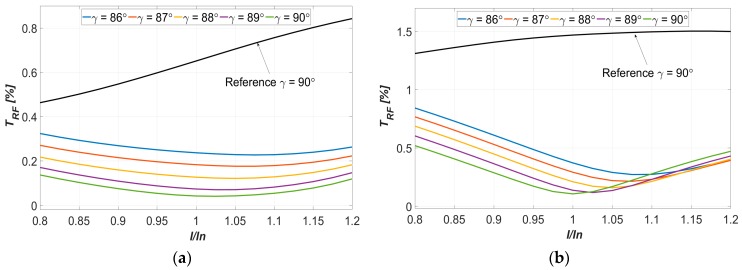
Torque ripple vs. relative current characteristics for different values of the torque angle γ (**a**) machine no. 1 of *n_s_*/*n_p_* equal to 9/10 and (**b**) machine no. 2 of *n_s_*/*n_p_* equal to 9/8.

The optimization of the supply current harmonics was carried out for the rated operational conditions of the studied machines (i.e., rated speed, torque and current values). In practice, the PMSM machines usually work with variable torque loads (corresponding to the supply current value), as well as the torque angle γ, which can be slightly different to 90°. To assess the robustness of the proposed approach, the torque ripple factor was determined for the current value in the range from 0.8 to 1.2 of the rated current and γ in the range from 86° to 90°. The characteristics of *T_RF_*, obtained as a function of the relative current, have been compared to the characteristics of the reference sinusoidal supplied machine (i.e., the machine without harmonic injection); see Figure 8a,b, respectively, for the machines with 9/10 and 9/8 *n_s_*/*n_p_* ratio.

As can be noted, the results obtained show that for all the considered conditions (i.e., currents and γ values), when the determined current harmonics are injected, both machines exhibit a significantly lower level of torque ripples. Simulations also show that further optimization of the injection of the current harmonics is possible. Extending the design space using the values of the current amplitude and the torque angle would allow for the definition of the amplitudes of particular harmonics as nonlinear functions of the fundamental harmonic amplitude and torque angle. Consequently, the suppression of the torque ripples will be more effective across the whole range of the torque demanded of the machine.

### 4.3. Infuence of Triplen Harmonics

**Figure 9:**
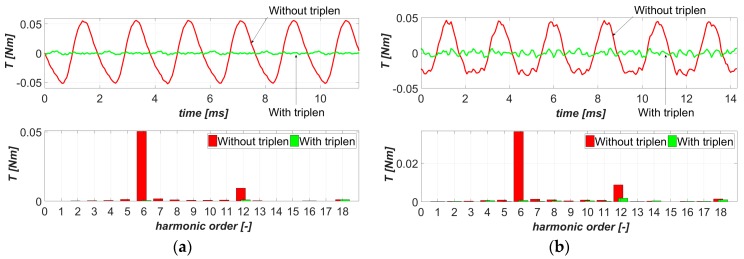
Comparisons of torque ripple waveforms and their harmonic contents, illustrating triplen current harmonics’ impact for (**a**) machine no. 1 with *n_s_*/*n_p_* equal to 9/10, and (**b**) machine no. 2 with *n_s_*/*n_p_* equal to 9/8.

In the last stage, the impact of triplen harmonics was examined. The calculations were performed for the optimized current shape (i.e., determined harmonics phases and amplitudes, including triplen harmonics), in case (a) and, in case (b), for the phase current waveforms without triplen harmonics, i.e., *I_P_*_3_ = *I_P_*_9_ = *I_P_*_15_ = 0. The torque ripple waveforms and their harmonic contents for both machines studied were compared in Figure 9a,b, respectively.

It can be noted that triplen harmonics have a significant influence on the effectiveness of torque ripple minimization by current harmonic injection. When comparing the *T_RF_* values, the lack of triplen harmonics increases the torque ripple level from 0.04% to 1.02% for the first machine, and from 0.11% to 0.88% for the machine with an *n_s_*/*n_p_* ratio equal to 9/8. It should be noted that the elimination of the triplen harmonics in machine no. 1 causes an increase in torque ripples above the level of the reference supply conditions (i.e., fundamental harmonic current). This indicates that, for the three-leg inverter-powered PMSMs, the current shape optimization should be carried out as an independent research task, performed prior to assuming a lack of triplen harmonics.

## 5. Conclusions

In this paper, a method for torque ripple minimization by current modulation in a PMSM drive has been presented and discussed. In the proposed approach, to find the optimal shape of the phase current waveforms, the torque ripple level is evaluated by means of a detailed numerical model of the magnetic field inside the machine, ensuring the results obtained are highly reliable. Despite higher computational complexity, in relation to approaches based on analytical models, the utilization of FEM based models allows the saturation phenomena, as well as other parasitic effects occurring in PMSMs, to be taken into account. 

To demonstrate the universality and effectiveness of the method, an analysis of the impact of the phase current harmonic injection on the electromagnetic torque ripple level of the permanent magnet synchronous machine driven by the six-leg inverter was carried out. Two FSCW PMSMs of SPM rotor structure and different numbers of poles were examined as a case study problem. To reduce the level of torque ripples, the waveforms of the phase currents were modulated by higher harmonics. A wide range fractional analysis was performed. The results obtained prove the effectiveness of the method, showing that the smoothness of the torque waveform can be significantly improved by the current harmonic injection. Comparing the value of the torque ripple factor of the machine supplied by the reference sinusoidal current (0.65%) to *T_RF_* for the optimized current shape (0.04%), it can be concluded that the torque ripple level in the studied PMSM with an *n_s_*/*n_p_* ratio equal to 9/10 was reduced by more than 16. In the case of the second tested PMSM, the torque ripples were reduced by around 13. Simulations also show that the torque ripple level depends on the current value (consequently, the effective torque value) as well as the torque angle. Nevertheless, the results obtained show that for all considered conditions (i.e., variation of the currents and torque angle γ values), when the determined current harmonics are injected, both machines studied exhibited significantly lower levels of torque ripples than the reference sinusoidal supplied machines.

Our ongoing task is to practically validate the effectiveness of the current harmonic injection method on the PMSM test drive supplied by a six-leg inverter. The results obtained will provide the scope for further publications by the authors.
